# First detection of a *Sesamia nonagrioides* resistance allele to Bt maize in Europe

**DOI:** 10.1038/s41598-018-21943-4

**Published:** 2018-03-05

**Authors:** Ana M. Camargo, David A. Andow, Pedro Castañera, Gema P. Farinós

**Affiliations:** 1 Department of Environmental Biology, Centro de Investigaciones Biológicas, CSIC, Madrid, 28040 Spain; 2 Department of Entomology, University of Minnesota, Saint Paul, MN 55108 USA

## Abstract

The Ebro Valley (Spain) is the only hotspot area in Europe where resistance evolution of target pests to Cry1Ab protein is most likely, owing to the high and regular adoption of Bt maize (>60%). The high-dose/refuge (HDR) strategy was implemented to delay resistance evolution, and to be effective it requires the frequency of resistance alleles to be very low (<0.001). An F_2_ screen was performed in 2016 to estimate the frequency of resistance alleles in *Sesamia nonagrioides* from this area and to evaluate if the HDR strategy is still working effectively. Out of the 137 isofemale lines screened on Cry1Ab maize leaf tissue, molted larvae and extensive feeding were observed for two consecutive generations in one line, indicating this line carried a resistance allele. The frequency of resistance alleles in 2016 was 0.0036 (CI 95% 0.0004–0.0100), higher but not statistically different from the value obtained in 2004–2005. Resistance does not seem to be evolving faster than predicted by a *S. nonagrioides* resistance evolution model, but the frequency of resistance is now triple the value recommended for an effective implementation of the HDR strategy. Owing to this, complementary measures should be considered to further delay resistance evolution in the Ebro Valley.

## Introduction

The commercial use of genetically engineered (GE) crops in Europe has been controversial. The only GE crop allowed for cultivation in the European Union (EU) is MON 810 maize, which expresses the *Bacillus thuringiensis* toxin Cry1Ab (Bt maize). Although Bt maize was sown in four EU countries in 2016, most of it was in Spain (94.7%), the only European country where Bt maize has been grown steadily since 2003^1^. More specifically, Bt maize farming is largely concentrated in the Ebro Valley, located in northeast Spain, where over 50% of all maize sown since 2007 expresses the toxin Cry1Ab^2^. The intensive cultivation of Bt maize coupled with the presence of several generations per year of the target pests *Sesamia nonagrioides* Lefèbvre (Lepidoptera: Noctuidae) and *Ostrinia nubilalis* Hübner (Lepidoptera: Crambidae), render the Ebro Valley as the only hotspot in Europe where resistance might evolve^3^. Owing to this, use of adequate insect resistance management strategies is key to ensuring the long-term sustainability of Bt maize in Spain.

The strategy known as high-dose/refuge (HDR) has been widely adopted to delay evolution of resistance to Bt crops^4,5^. This strategy is based on the use of high-dose Bt maize varieties that eliminate most heterozygotes in the pest population, along with the sowing of non-Bt varieties close to Bt fields that act as refuges for susceptible individuals. For the HDR approach to be effective, mating should be random within fields, so that resistant individuals emerging in a Bt field will most likely mate with individuals from the larger susceptible refuge population, resulting in heterozygous offspring that are susceptible and therefore killed by Bt plants. Other requirements for this strategy to work effectively are recessive inheritance of resistance and a very low frequency of resistance alleles, ideally <0.001^6–8^.

Since Bt crops were first commercialised in 1996, several cases of field-evolved resistance have been reported worldwide^5^. The evolution of resistance has often been the result of failure to meet the requirements of the HDR strategy, including poor refuge compliance^9–12^ and use of non-high dose events^10^. Non-recessive inheritance of resistance might have also contributed to control failure in the case of *Diabrotica virgifera virgifera* resistant to Cry3Bb1 maize in the US^10^. Additional environmental factors may have promoted the evolution of resistance in the field^13^. For instance, the tropical climate in both Puerto Rico and northern Brazil allowed for continuous cropping and overlapping generations of *Spodoptera frugiperda* throughout the entire year, which would have resulted in repetitive strong selective pressure on the pest and contributed to rapid control failure of Cry1F maize in both countries^11,14^. Pest preference for irrigated Bt fields versus non-irrigated refuges could have also played a role in some cases of resistance evolution^9,14^.

A study carried out in 2004–2005 used an F_2_ screen to estimate the frequency of resistance alleles to Cry1Ab maize in populations of *S. nonagrioides* from the Ebro Valley, where it is the most harmful maize pest, and Greece^15^. Previous works had suggested this stem borer species was a single panmictic unit in Southern Europe^16^, leading to an estimated expected frequency of resistance of 0.0015, very close to the low frequencies required for the HDR strategy to be effective^15^. This value was based on a relatively small number of samples, and there has been a steep increase in the adoption of Bt maize in the Ebro Valley, from 35% in 2005 to 74% in 2016^2^, so it is important to re-examine the frequency of resistance. Consequently, the resistance allele frequency was estimated here to evaluate if the resistance risk has changed and assess if the assumption of low resistance frequency for the HDR strategy still holds. The findings of this work will help to elucidate whether the HDR approach is working effectively in this area or the resistance management strategy should be revised.

## Results

A total of 1,327 fifth and sixth instar larvae of *S. nonagrioides* were collected in non-Bt maize fields at four regions of the Ebro Valley in September and October of 2016 (Table 1). Upon emergence, 385 pairs of adults were confined in single-pair cages for mating and egg-laying. One hundred and fifty-four of these pairs (40.0% of the initial number) produced enough fertile eggs and went on to establish isofemale lines, whereas the remaining pairs did not mate or produced few viable eggs. Sib-mating of the F_1_ occurred in 143 of the lines (37.1%), and 137 of them (35.6%) produced enough viable offspring as to allow for an F_2_ screen (Table 1). The average F_1_ family size in the lines that were screened was 27.4 ± 1.0 females and 27.2 ± 1.0 males, and an average of 11.4 ± 0.6 neonates per F_1_ female and line were screened on Bt leaf tissue (mean ± s.e.m.). Average fecundity per female was 668.4 ± 14.2 in the parental generation and 230.5 ± 8.0 in the F_1_ generation (mean ± s.e.m.). A Mann-Whitney U test was carried out to determine if differences in fecundity between generations were significant. Results of this analysis showed that there was a significant reduction in *per capita* fecundity between the parental generation and the F_1_ generation in the 137 lines that were subjected to an F_2_ screen (U = 240; p < 0.001).

**Table 1:** Number of larvae collected in each region and number of isofemale lines progressing through each step of the F_2_ screen.

Region	Number of larvae collected	P_0_ lines established	Lines that produced F_1_ larvae	Lines that produced F_1_ adults	F_2_ screened
Los Monegros	410	126	55	52	50
Bajo Cinca	493	147	63	61	60
Tafalla	387	101	33	32	26
Valdejalón	37	11	3	2	1
TOTAL	**1,327**	**385**	**154**	**147**	**137**

**Figure 1:**
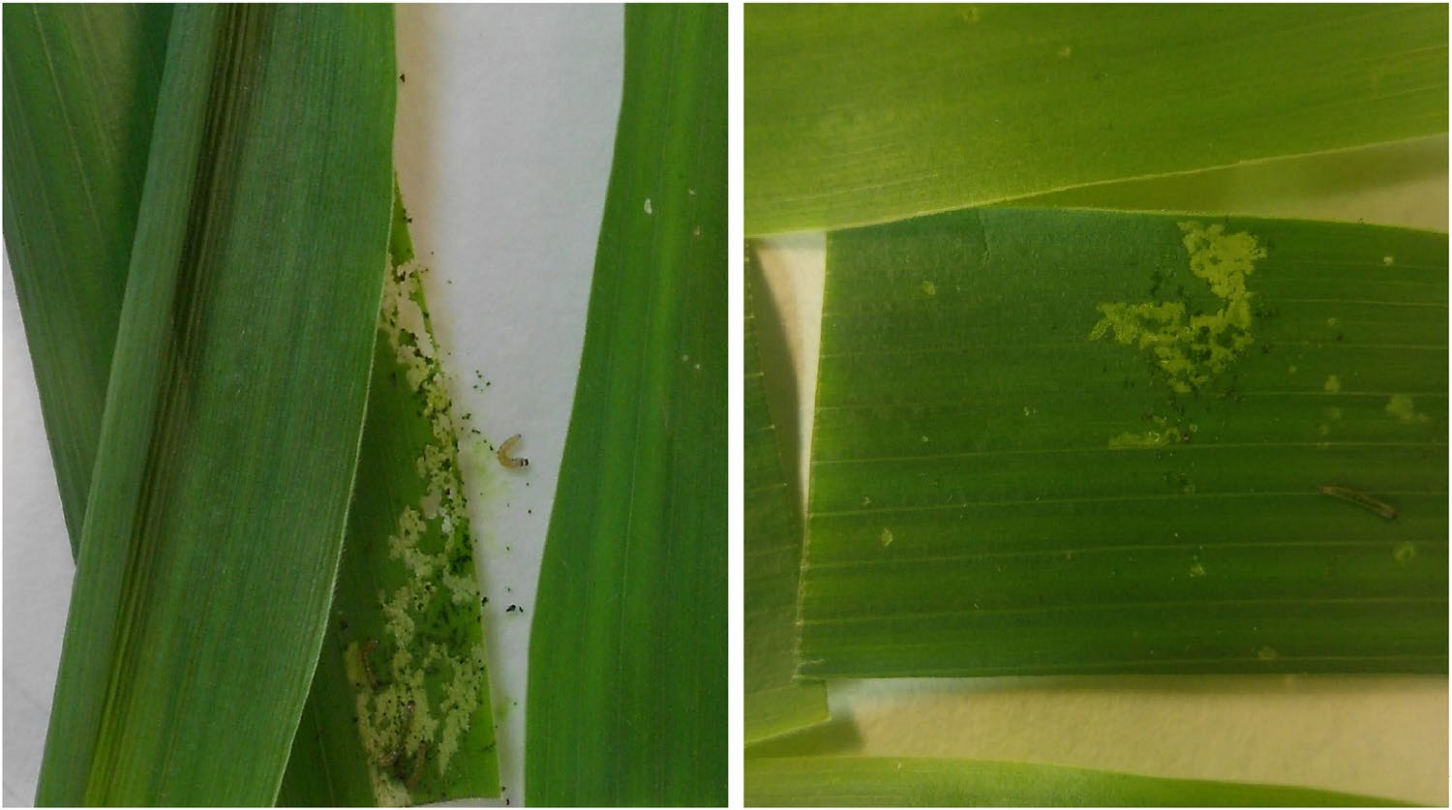
Leaf damage in Bt leaf tissue caused by larval feeding in the F_3_ screen. Extensive larval feeding and larvae of the 2^nd^ instar were detected in Bt maize on day 5 of the rescreen of the F_3_ of line P350.

Larval survival after 5 days of exposure to Bt maize was observed in 104 of the 137 screened lines (75.9% of the lines tested). However, molted larvae and extensive leaf damage due to larval feeding were only detected in line P350, which consequently was considered the only potentially positive line. To confirm the presence of a major resistance allele, this line was rescreened using F_3_ neonates. Substantial feeding damage and second-instar larvae were detected on fresh Bt leaves on day 5 of the rescreen (Fig. 1), rendering P350 as a true positive line. Control mortality in conventional maize dishes was 13.2 ± 0.8% in the F_2_ screen, and 2.3% in the F3 screen in line P350 (mean ± s.e.m.).

Considering that one true positive line was detected (S = 1) after screening N = 137 lines, the expected frequency of major resistance alleles (q) was estimated to be E(q) = 0.0036, with a 95% credibility interval between 0.0004 and 0.0100.

**Figure 2:**
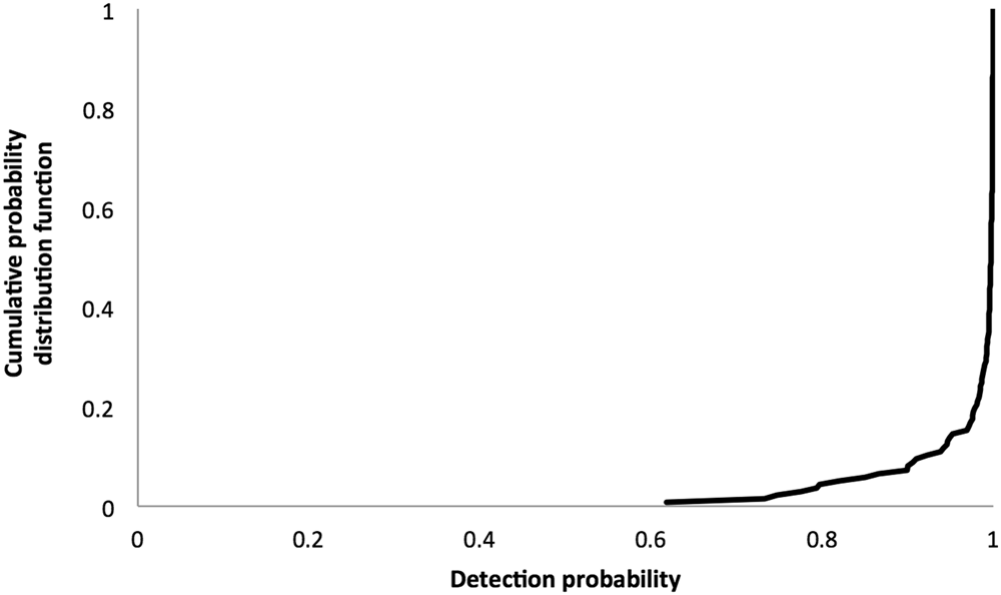
Cumulative probability distribution function (CDF). Cumulative probability of detecting a resistance allele, calculated as 1-P_No_, where P_No_ is the type II error (not detecting a resistance allele that is present in the line). Experiment-wise detection probability was 97.5%, and 86.1% of the lines had a detection probability >95%.

As shown in Fig. 2, the probability of detecting a resistance allele (1-P_No_) was >95% in 86.1% of the lines tested, and only 4.4% of the lines had a detection probability <80%. The experiment-wise detection probability was 97.5%, meaning that if a resistance allele was present, it would have been detected 97.5% of the times the experiment was performed. This value might be an underestimate of detection probability, since control mortality was recorded at day 8 and it is likely lower values would have been detected if it had been recorded at day 5, just like mortality in Bt maize was. Data necessary to calculate P_No_ can be found in Supplementary Table S1.

To assess whether expected frequency of the resistance allele (R) had changed since it was estimated in *S. nonagrioides* populations in 2004–2005^15^, the joint probability density function of these estimates was calculated. Results of this test indicate the two estimates were not statistically different, although the probability that they were the same was only p = 0.21. Results of a simulation of 10,000 replications that considered the beta distribution of both estimates concluded significant differences would have been found if twice the number of lines had been screened in 2004–2005, whereas screening a larger number of lines in 2016 would not have changed this result.

When random samples from the estimated probability distribution of the initial R allele frequency were used to initialize the evolutionary model^15^, they poorly predicted the probability distribution of the R allele in 2016 (Supplementary Fig. S1). This indicated that the probability distribution of the initial R allele frequency was poor. We then used the expected value of the initial R frequency^15^ to predict the 2016 R frequency. The observed expected frequency (0.0036) was not significantly different from the predicted frequency (0.0033, p-value = 0.12), although it was higher than predicted (Supplementary Fig. S2). Consequently, the evolutionary model was reinitialized with the 2016 R allele frequency to predict the number of years to resistance from 2016. The updated model predicted resistance would occur in 31 years from 2016, which was 2.8 years earlier than predicted with the 2004–2005 R allele frequency^17^.

## Discussion

For two consecutive generations larvae of line P350 molted to second instar and caused substantial feeding damage after 5 days on Bt leaf tissue, indicating that this line carried a resistance allele. To our knowledge, this is the first time a resistance allele has been detected in a field population of *S. nonagrioides*. Moreover, no previous works have reported a reduction in susceptibility to Cry1Ab toxin in field populations of this species, either in the Ebro Valley or elsewhere^17,18^.

The ability of neonate *S. nonagrioides* of line P350 to provoke a “light defoliation” on Bt leaves is “practical resistance” as defined by Tabashnik *et al*.^19^. Susceptible larvae are unable to cause light defoliation on Bt plants. This “light defoliation” could reduce Bt plant efficacy and have practical consequences for pest control. Newly hatched larvae of this species only need to eat a small amount of maize leaves, because they can then tunnel into the stalk where they feed until pupation. In stalks, the expression of the toxin is lower^20^. Once they reach the stalk, they could feed on the stem and cause yield losses that could be particularly damaging in the case the first generation larvae, when maize is in the early stages of development.

The sharp decrease in female fecundity between the parental and first generation probably did not bias the R allele frequency estimates. If lines carrying a resistance allele were selectively lost or retained prior to the screen, our 2016 estimate would be biased^21^. However, even the reduced number of F_1_ eggs was more than sufficient to provide a high detection probability (97.5%). The decrease probably was not due to inbreeding because the effects of inbreeding would only occur during the F_2_ generation. The low values of mortality on conventional maize observed in both the F_2_ and F_3_ screens further indicate the absence of strong inbreeding depression in the isofemale lines. Instead, the decrease in fecundity was most likely related to the high larval density experienced by larvae in the F_1_, which has been observed to be associated with smaller pupae and lower fecundity in *S. nonagrioides*^22^. Moreover, the parental generation comes from larvae that were in diapause, and diapausing larvae of *S. nonagrioides* undergo several supernumerary moults without pupating^23,24^, resulting in larger and consequently more fecund adults compared with non-diapausing adults^25^.

The results of our experiments suggest that the frequency of resistance increased slightly from 2004–2005 to 2016 in the Ebro Valley, a difference that would have been statistically significant had the 2004–2005 estimate been more thorough. Our model predicts that resistance frequency should increase only slightly by 2016 from the initial estimate during 2004–2005^17^. This slight increase is probably related to the strong selective pressure posed by the high adoption rate of Bt maize in the Ebro Valley for most of the last decade^2^. Additionally, use of Bt varieties containing Event 176 between 1998 and 2005, which expressed a lowering toxin titer as the season progressed, could have accelerated the evolution of resistance^17,26^. Overall, these results emphasize the importance of continued careful monitoring of resistance evolution in the Ebro Valley and the need to consider using additional strategies to slow down this process.

Although the frequency of R has increased slightly, resistance is not evolving faster than expected, implying that the HDR strategy may continue to be effective at delaying the evolution of resistance to Bt maize in *S. nonagrioides* in the Ebro Valley. However, the frequency of resistance in 2016 (0.0036) is three times the recommended value for implementation of the HDR approach (<0.001), which reduces the expected time to resistance failures (31 years from 2016). Now that a resistance allele has been detected, it is a matter of time before homozygous resistant individuals emerge and start damaging Bt fields.

The use of pyramided maize varieties that express several *B. thuringiensis* toxins would be a natural step towards delaying resistance evolution^4,27^. These varieties should be carefully designed to combine toxins with low probability of cross-resistance between them, so as to increase their effectiveness^28,29^. However, the longer evolution proceeds against Cry1Ab Bt maize, the more likely a pyramid with Cry1Ab will be merely sequential use of two toxins, eliminating the advantages of a pyramid for resistance management^30^. Pyramided Bt maize varieties are not available for cultivation in the EU. The approval of new GE crop varieties for cultivation in the EU is a lengthy and complex process that ultimately requires a majority of Member States to vote in favor of them^31,32^. At this time, however, a growing number of European countries have stopped or even banned cultivation of GE crops in their territories^1,33^ and there is a strong public opposition to genetically modified crops in the EU^34^. Hence despite the substantial advantages of pyramided varieties for resistance management, it seems unlikely that they will be available in the EU anytime soon.

Alternatively, increasing the percentage of the crop allotted to conventional maize, that is, increasing refuge size, could reduce the selection pressure exerted on the pest by promoting disassortative mating between susceptible individuals coming from refuges and insects carrying resistance alleles. This would help to increase heterozygosity^35^, thus lowering the frequency of homozygous resistant individuals. This approach has been suggested to manage field-evolved resistance to Cry3Bb1 maize in Western Corn Rootworm in the US^36^.

In conclusion, this is the first work that reports detection of resistance to a Bt crop in the EU, specifically to Cry1Ab maize in *S. nonagrioides*. The frequency of resistance alleles has increased slightly in populations of this pest in the Ebro Valley in northeast Spain, although this is not happening faster than predicted by the *S. nonagrioides* resistance evolution model. According to the updated version of this model, control failure is predicted to happen in 2047. Finally, although the HDR strategy appears to have worked so far in managing evolution of resistance in this pest, the adoption of additional measures would ensure the long-term sustainability of Bt maize in the control of *S. nonagrioides* in this area.

## Methods

As outlined in Andow and Alstad^21^, an F_2_ screen is a four-step method, consisting of (1) sampling of individuals in the field and establishment of isofemale lines; (2) rearing of the F_1_ and sib-mating of the adults in each line; (3) testing susceptibility of each line to Bt toxins by screening the F_2_ neonates, and (4) statistical analysis of results. Given that each mated female carries four gametic haplotypes, if one of the parents carried a resistance allele, 1/16 (6.25%) of the F_2_ offspring would be expected to be homozygous resistant and test positive in the screen.

### Insect collection and establishment of isofemale lines

**Figure 3:**
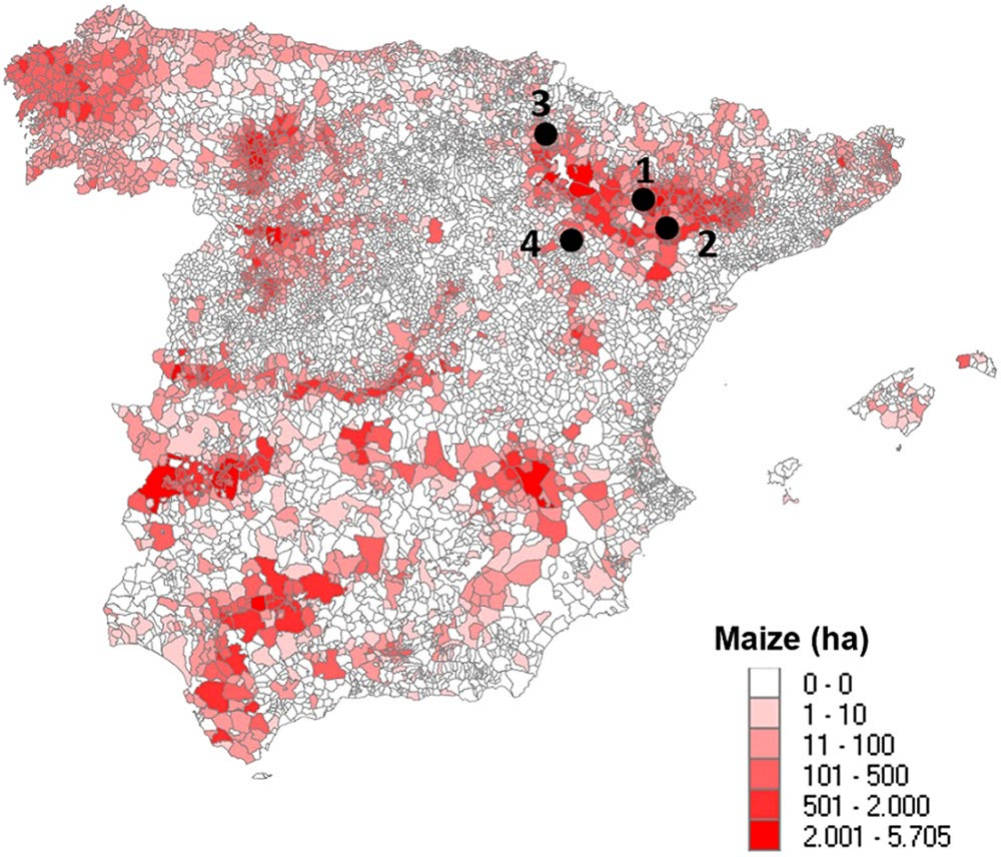
Cultivated surface (ha) of maize in municipalities of Spain. Sampling locations in the Ebro Valley are indicated by dots: Los Monegros (1), Bajo Cinca (2), Tafalla (3) and Valdejalón (4). The map has been generated by own compilation with data corresponding to the Agrarian Census 2009 (http://www.ine.es/dyngs/INEbase/en/operacion.htm?c=Estadistica_C&cid=1254736176851&menu=resultados&secc=1254736194950&idp=1254735727106), taken from the Instituto Nacional de Estadística (INE) website: www.ine.es. To generate the map the following free specific software was needed: PC-Axis 2008, PX-Map 2001, and Municipal Maps (outlines of municipalities valid at 1 January 2009). All this software is available on the INE website (http://www.ine.es/ss/Satellite?c=Page&p=1254735116596&pagename=ProductosYServicios%2FPYSLayout&cid=1254735116596&L=1).

Fifth and sixth instar larvae of *S. nonagrioides*, most of which had entered diapause, were collected in September and October of 2016 in four different regions of the Ebro Valley, Spain (Fig. 3): Los Monegros and Bajo Cinca in the province of Huesca, Tafalla in the province of Navarra and Valdejalón in the province of Zaragoza (Supplementary Text). Larvae were collected in 1–4 non-Bt maize fields at each location by dissecting damaged maize stalks, and placed in plastic boxes containing fresh pieces of maize stalks for transportation to the laboratory.

Upon arrival to the laboratory, larvae were surface sterilized by dipping in a 1% bleach solution for approximately 30 seconds and then allowed to dry. Groups of ≈50 larvae from the same field were transferred to plastic boxes and reared on a meridic diet^37^ on top of filter paper and a bottom layer of vermiculite to facilitate pupation. Boxes were stored in growth chambers (SANYO, MLR-352 PE, Japan) at a temperature of 16 ± 0.3 °C and a 12:12 L:D photoperiod to maintain diapause. Every 3–4 days fresh diet was added and every box was examined for pupae.

When an increase in pupation was observed, environmental conditions were shifted to 25 ± 0.3 °C and continuous light to promote breaking of diapause. Pupae were separated according to their sex and field of collection and kept in plastic boxes until adult emergence. Each emerging adult was paired individually with an adult of the opposite sex originating from the same location, but not necessarily from the same field. Oviposition cages consisted of two maize seedlings placed in a cylindrical plastic cup and confined by a ventilated plastic cup on top. Egg masses were collected seven days later and placed on top of moistened filter paper in plastic boxes (8.9 cm diameter × 2.3 cm height).

### Rearing and sib-mating of the F_1_

The offspring of each two-parent family defined one line and were reared separately on meridic diet, first in 11.5 cm diameter × 4.5 cm height boxes and later in 23 × 21 × 5 cm boxes. When the last larval stage was reached, vermiculite was added to the bottom part of the boxes for pupation. Pupae were collected and their sex was determined. Upon adult emergence, a single oviposition cage consisting of a pot with 25 maize seedlings confined by a ventilated plastic cylinder (20 cm diameter x 45 cm height) was set up per isofemale line for sib-mating of the F_1_ adults, and the number of females and males placed in each cage for 2–3 consecutive days was recorded. Egg masses were collected seven days later and placed on moistened filter paper. In each line, the number of eggs laid in both the parental and first generations was estimated. Rearing and mating of the F_1_ generation took place under a temperature of 25 ± 0.3 °C and a photoperiod of 16:8 L:D.

### Testing susceptibility to Cry1Ab maize

In each line 60–250 neonates (<24 hours old) were placed in ventilated plastic dishes (8.9 × 2.3 cm) containing 4–5 pieces of Bt maize leaves from which the mid-rib had been removed, to prevent larvae from tunneling inside and avoiding or reducing exposure to Cry1Ab toxin. Leaves were excised from Bt maize plants (DKC 4796YG, Monsanto, St. Louis, USA) at the V6–10 phenological stage. Before the screens were performed the presence of Cry1Ab toxin was confirmed in every plant using lateral flow test strips (ImmunoStrip® for Bt-Cry1AbProtein/Bt-Cry1Ac-Protein, AGDIA Biofords, France). Moistened filter paper was added to the dishes to keep leaves turgid and every 2–3 days fresh leaves were added to each dish. Larval survival and leaf damage were evaluated on day 5 of the screen. Larvae that did not move when touched with a fine hair brush were considered dead. A line was considered positive if extensive feeding damage and second instar larvae were detected in Bt maize assay dishes on day 5. Additionally, control dishes with 20–50 neonate larvae from a line were screened on conventional maize (DKC4795, Monsanto, St. Louis, USA) and mortality was recorded 8 days later. All screens were performed at 25 ± 0.3 °C and a 16:8 L:D photoperiod.

Lines that tested positive in the F_2_ screen were rescreened following the same procedure in the next generation to confirm the presence of a resistance allele. For this purpose, larvae that were not used in the F_2_ screen were reared to produce the third generation. A line that tested positive in both the second and the third-generation screens was considered to be a true positive and thus to carry a resistance allele.

### Statistical analyses

Bayesian statistics, which allow statistical inferences about the studied population, were used to analyze the data. The expected frequency of resistance alleles [E(q)] and its 95% credibility intervals were calculated according to equations in^21,38,39^. Mathematica 8.0 (Wolfram Research, 2011) was used to calculate these parameters.

Variation in female fecundity between generations was studied to evaluate possible inbreeding depression. The number of eggs laid per female in the first and second generations of each line was estimated from photographs, counting eggs individually in the parental generation and using GIMP 2.8.20 software for the first generation. In the latter case, the digital images were processed to distinguish eggs from the whitish background. For this purpose, the blue channel of a split RGB image was used, and the number of pixels with an intensity between 0 and 98 (0 = black; 255 = white) represented eggs. The number of eggs corresponding to a given number of pixels was estimated by regression, and the regression equation was used to estimate egg numbers for each female. As the distribution of fecundities was non-normal, a Mann Whitney U test was used to compare per capita fecundity between generations.

The probability of missing a resistance allele (false negative) that was present in an isofemale line (P_No_) was estimated for each line as a function of the number of F_1_ males and females that produced the F_2_ neonates, the number of screened neonates per F_1_ female, and the average control mortality on conventional maize (µ)^39^. Calculations were performed using R 3.4.0. Detection probability of each line was calculated as 1-P_No_, and the overall detection probability of the experiment was estimated as an average of the values of all lines.

To ascertain if the expected frequency of resistance estimated from this sample differed from the initial estimate obtained from 2004–2005^15^, the joint probability density function for the two estimates was calculated as described in Wenes *et al*.^40^. When p > 0.05, the estimates were considered not statistically different. Mathematica 8.0 (Wolfram Research, 2011) was used for this analysis.

The *S. nonagrioides* resistance evolution model^17^ was updated to include data on Bt adoption rate for the years 2014–2016. Two simulations of 300,000 runs were conducted to predict the resistance frequency in 2016: first initializing the R allele frequency randomly from the posterior beta distribution of the 2004–2005 estimate^15^ and second, initializing it with the expected value for 2004–2005^15^. Finally, the *S. nonagrioides* resistance evolution model was updated to use the 2016 estimated resistance frequency as the initial value and recalculate the number of years to resistance failure. Large differences compared with the previous estimate^17^ would indicate resistance is not evolving as originally predicted. Mathematica 8.0 (Wolfram Research, 2011) was used to perform these simulations.

### Data availability

All data generated or analysed in this study are included in this published article (and its Supplementary Information file).

## Electronic supplementary material

Supplementary information
