# Supplementary material for: First detection of a Sesamia nonagrioides resistance allele to Bt maize in Europe
Source: Sci Rep. 2018 Mar 5;8:3977. doi: 10.1038/s41598-018-21943-4 (PMC5838095; doi:10.1038/s41598-018-21943-4)
Supplement: Supplementary file 1 — Supplementary information [file 41598_2018_21943_MOESM1_ESM.pdf]

SUPPLEMENTARY INFORMATION

First detection of a *Sesamia nonagrioides* resistance allele to Bt maize in Europe

Ana M. Camargo, David A. Andow, Pedro Castañera and Gema P. Farinós

Supplementary Table S1. Type II error (P_{No}) was calculated for each line that was subjected to the F_2 screen based on the number of F_1 females and males and the number of F_2 neonates screened on Bt maize per female. Only 385 lines were part of the experiment even though 418 pairs of adults were initially in the parental generation, as 33 lines were randomly discarded during the first stage of the F_1 generation due to logistical issues.

Line number	F_1 females (F)	F_1 males (M)	Number of neonates screened per F_1 female (J)	Control mortality (%)
**6**	23	34	10.9	12
**9**	31	43	8.1	4
**12**	11	7	22.7	24
**13**	26	22	9.6	0
**18**	20	34	12.5	0
**20**	8	10	31.3	10
**22**	33	33	7.6	22
**27**	34	28	7.4	18
**28**	26	36	9.6	10
**29**	27	24	9.3	10
**31**	24	26	10.4	14
**33**	28	24	8.9	26
**34**	17	14	14.7	26
**38**	27	32	9.3	28
**40**	16	24	15.6	12
**44**	35	26	7.1	8
**45**	7	9	14.3	20
**46**	21	20	11.9	2
**49**	24	10	10.4	0
**50**	14	28	17.9	14
**51**	5	9	50.0	10
**55**	26	25	9.6	32
**59**	27	36	9.3	4
**67**	26	13	9.6	16
**68**	7	5	24.3	32
**74**	35	25	7.1	14
**78**	27	20	9.3	30
**79**	15	15	16.7	38
**93**	11	26	22.7	0
**100**	36	55	6.9	18
**104**	41	38	6.1	10
**106**	28	39	8.9	8
**108**	35	42	7.1	18
**109**	36	46	6.9	42
**112**	15	7	16.7	4
**113**	17	21	14.7	6
**115**	18	21	13.9	8

**123**	32	39	7.8	10
**124**	32	24	7.8	20
**126**	40	26	6.3	16
**129**	38	25	6.6	12
**132**	26	30	9.6	12
**133**	8	7	31.3	8
**134**	54	47	4.6	6
**137**	29	27	8.6	12
**138**	29	25	8.6	6
**139**	12	10	20.8	24
**141**	22	12	11.4	4
**145**	37	37	6.8	22
**150**	26	35	9.6	26
**153**	39	50	6.4	8
**156**	36	52	6.9	16
**162**	23	25	10.9	10
**164**	40	34	6.3	10
**165**	36	29	6.9	18
**167**	17	12	14.7	60
**168**	37	34	6.8	6
**171**	26	29	9.6	26
**172**	29	31	8.6	10
**173**	60	55	4.2	0
**176**	36	25	6.9	28
**189**	10	15	25.0	2
**190**	22	20	11.4	6
**191**	22	35	11.4	16
**192**	40	22	6.3	24
**194**	22	30	11.4	12
**196**	26	35	9.6	8
**201**	23	20	10.9	2
**204**	24	24	10.4	12
**205**	31	26	8.1	14
**210**	23	31	10.9	0
**211**	7	3	35.7	4
**212**	7	7	8.6	12
**216**	40	33	6.3	8
**218**	37	41	6.8	10
**220**	35	26	7.1	4
**226**	15	22	16.7	36
**229**	12	11	20.8	20
**232**	54	28	4.6	22
**236**	31	32	8.1	16
**237**	43	53	5.8	6
**247**	44	28	5.7	8
**255**	35	25	7.1	10
**256**	39	37	6.4	12
**260**	38	43	6.6	8
**261**	9	17	27.8	6
**263**	10	3	16.5	4
**266**	19	23	13.2	2
**268**	22	15	11.4	10

**269**	38	22	6.6	18
**272**	18	28	13.9	14
**274**	21	27	11.9	4
**276**	35	34	7.1	14
**277**	29	31	8.6	10
**278**	28	28	8.9	0
**289**	17	12	14.7	18
**296**	45	37	5.6	28
**297**	36	43	6.9	14
**301**	54	52	4.6	20
**302**	17	16	14.7	14
**303**	18	24	13.9	10
**305**	10	9	25.0	0
**308**	29	36	8.6	4
**309**	38	33	6.6	24
**312**	22	11	11.4	4
**313**	12	18	20.8	6
**314**	47	26	5.3	12
**316**	27	20	9.3	6
**323**	26	22	7.6	14
**328**	57	27	4.4	16
**329**	33	29	7.6	24
**330**	23	28	10.9	8
**331**	42	28	5.6	22
**338**	49	63	5.1	16
**341**	12	15	20.8	6
**345**	14	18	17.9	6
**347**	11	6	22.7	14
**350**	37	48	6.8	18
**352**	22	37	11.4	16
**360**	17	20	14.7	16
**362**	19	35	13.2	16
**363**	44	38	5.7	0
**375**	32	42	7.8	4
**378**	32	25	7.8	14
**380**	34	30	7.4	6
**381**	25	30	10.0	22
**385**	4	4	15.0	15
**392**	45	33	5.6	0
**394**	9	13	27.8	12
**396**	63	40	4.0	8
**400**	26	38	9.6	30
**402**	23	21	10.9	16
**404**	24	46	10.4	4
**406**	42	46	6.0	32
**407**	40	32	6.3	14
**409**	26	34	9.6	2
**412**	26	26	9.6	6
**Mean**	27.4	27.2	11.4	13.2
**s.e.m.**	1.0	1.0	0.6	0.8

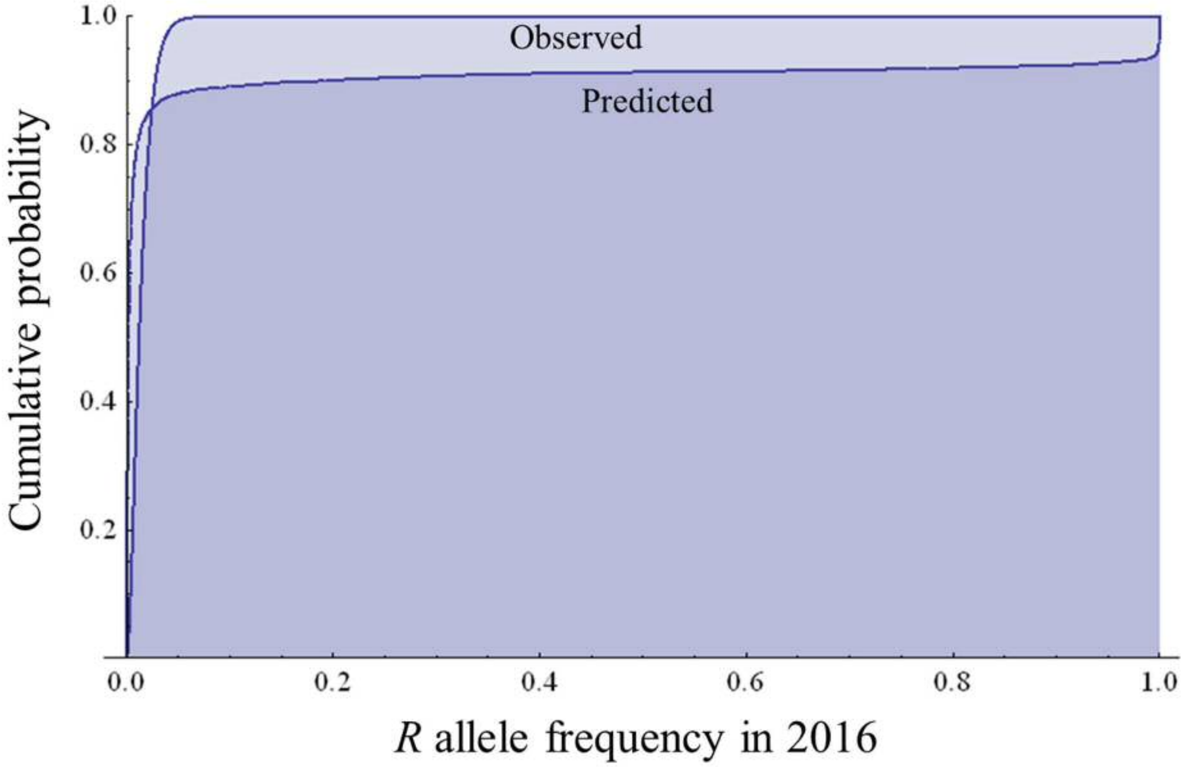

Supplementary Figure S1. Predicted and observed cumulative probability distributions for the R allele frequency in 2016. The observed distribution was based on the posterior beta distribution estimated in this paper. The predicted distribution was estimated from 10,000 Monte Carlo simulations of the *Sesamia nonagrioides* resistance evolution model[1], with initial R allele frequencies randomly selected from the beta distribution in Andreadis *et al*. (2007)[2] and updated with the area of Bt maize for 2014-2016. The two distributions are different, and the prediction is poor. The predicted distribution has about 5% of all cases with the R allele going to fixation by 2016 and about 10% of the cases the frequency is >0.5. Clearly, this has not happened. It also over-predicts the probability of a low R allele frequency in 2016. These results suggest that the initial R allele frequency distribution was poorly estimated, probably because only 160 F_2 lines were screened. This resulted in an initial probability distribution for the R allele frequency with a long tail, with significant probability of an R allele frequency >0.004, which was predicted to lead to rapid resistance failure.

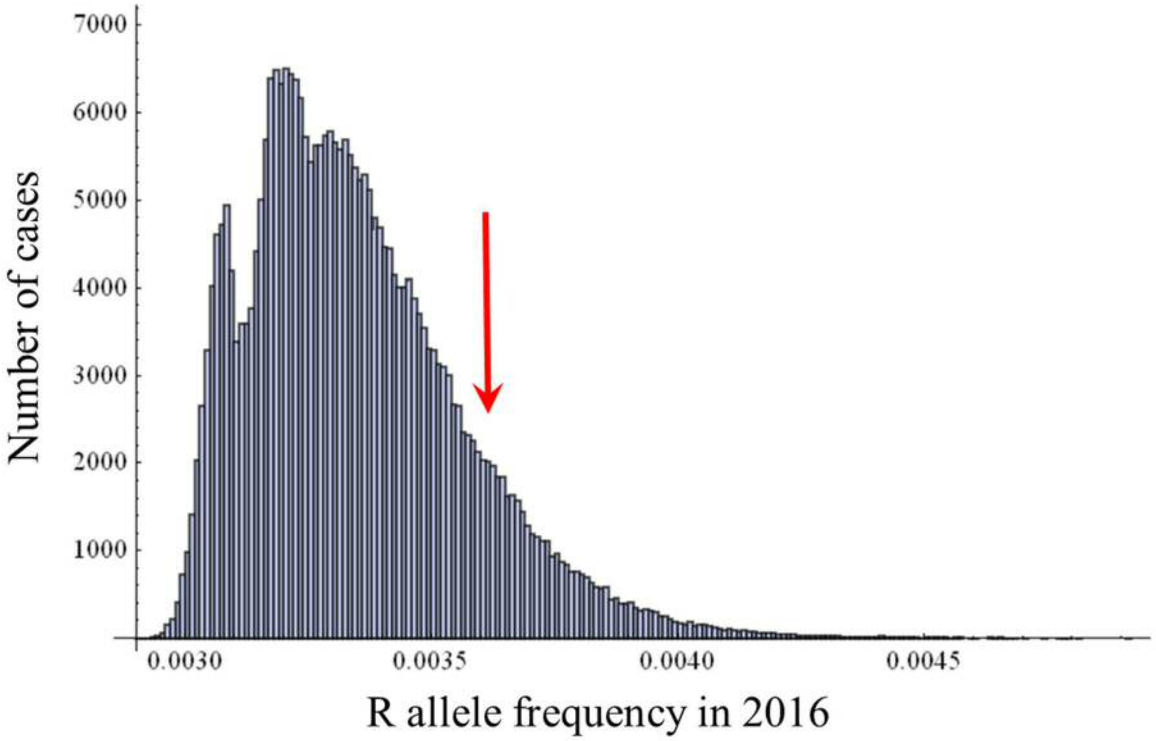

Supplementary Figure S2. Histogram of the predicted probability distribution for the *R* allele frequency in 2016. The predicted distribution was estimated from 300,000 Monte Carlo simulations of the *Sesamia nonagrioides* resistance evolution model[1], with the fixed initial *R* allele frequency = 0.0015 and updated with the area of Bt maize for 2014-2016. The mean of this distribution is 0.003347. Red arrow is the estimated *R* allele frequency estimated in this work (0.003617). The probability that the estimated frequency is from the predicted distribution is 0.1157 (ns). This suggests that the original estimated initial *R*-allele frequency may have been accurate. Although not statistically different, the current estimated frequency is higher than the predicted frequency for 2016.

Supplementary Text. As a geographic unit, the Ebro Valley includes parts or all of different Autonomous Communities in northeast Spain, the largest of which are Aragón and Cataluña. These two also are the major producers of maize in the Ebro Valley, where adoption of Bt maize reached 76.9% during 2016. The evolution of resistance in *Sesamia nonagrioides* was modeled in these two regions (Castañera *et al*. 2016). Recently, adoption of Bt maize has increased dramatically in Navarra, an Autonomous Community upstream and to the west of Aragón. The proportion of Bt maize in Navarra nearly tripled in the last decade, from 21% in 2004-2005 to 54% in 2016. Consequently, in this study, we have included Navarra as a part of the Ebro Valley. Navarra contributes ~15% (2015) of the maize area in the Ebro Valley.

References for Supplementary Information

1. Castañera, P., Farinós, G. P., Ortego, F. & Andow, D. A. Sixteen years of Bt maize in the EU hotspot: Why has resistance not evolved? PLoS ONE. **11**(5), e0154200.doi:10.1371/journal. pone.0154200 (2016).
2. Andreadis, S. S. *et al*. Frequency of resistance to *Bacillus thuringiensis* toxin Cry1Ab in Greek and Spanish population of *Sesamia nonagrioides* (Lepidoptera: Noctuidae). *J. Econ. Entomol*. **100**(1)**,** 195-201 (2007).
